# Digoxin-induced anemia among patients with atrial fibrillation and heart failure: clinical data analysis and drug-gene interaction network

**DOI:** 10.18632/oncotarget.18504

**Published:** 2017-06-16

**Authors:** Yubi Lin, Siqi He, Ruiling Feng, Zhe Xu, Wanqun Chen, Zifeng Huang, Yang Liu, Qianhuan Zhang, Bin Zhang, Kejian Wang, Shulin Wu

**Affiliations:** ^1^ Guangdong Cardiovascular Institute, Guangdong General Hospital, Guangdong Academy of Medical Sciences and Medical School of South China University of Technology, Guangzhou 510080, P.R. China; ^2^ The First Affiliated Hospital of Jinan University, Guangzhou 510630, P.R. China; ^3^ Division of Cardiac Surgery, First Affiliated Hospital of Sun-Yat-sen University, Guangzhou 510080, P.R. China; ^4^ Department of Biochemistry and Molecular Biology, Medical College, Jinan University, Guangzhou 510632, P.R. China; ^5^ Lin He's Academician Workstation of New Medicine and Clinical Translation at The Third Affiliated Hospital, Guangzhou Medical University, Guangzhou 510150, P.R. China

**Keywords:** digoxin, atrial fibrillation, heart failure, anemia, FDA adverse event reporting system

## Abstract

Digoxin is widely used to treat various heart conditions. In order to clarify the association between digoxin and anemia adverse reaction, we inspected case reports submitted to the FDA Adverse Event Reporting System (FAERS) between January 2004 and December 2015. These reports involved 75618 atrial fibrillation patients and 15699 heart failure patients. Compared to other therapies, digoxin treatment was significantly more likely to be concurrent with anemia adverse reaction among both atrial fibrillation patients (pooled OR = 1.38, 95% CI 1.14–1.68, P-value = 0.001) and heart failure patients (pooled OR =1.50, 95% CI 1.33–1.59–, P =4.27×10^−5^). We further explored previously published evidences and found 821 human genes directly or indirectly interacting with digoxin. Functional analysis indicated that these genes were significantly enriched in the biological processes of iron transport, which are closely related to iron deficiency anemia. Taken together, our retrospective analysis demonstrated the significant association between digoxin treatment and anemia adverse reaction, which should be seriously considered in clinical practice. Functional enrichment analysis on digoxin-related genes warranted subsequent research on the underlying toxicological mechanisms.

## INTRODUCTION

Drug administration may be responsible for toxic effects such as anemia. A long list of medications, including many commonly-used drugs, are associated with anemia. The frequency of anemia adverse events varies between drugs. In some cases, anemia can be associated with significant morbidity and mortality [1], which could pose a challenge in clinical treatment and nursing of many disorders. Prior knowledge of the risk for drug-induced anemia and monitoring the patients who are at risk are critical to ensure timely and appropriate response to potential adverse reactions [2].

Digoxin is a medication widely used to treat various heart conditions, such as heart failure [3] and atrial fibrillation [4]. The main pharmacological effects of digoxin on the heart involve inhibition of the sodium potassium adenosine triphosphatase (Na+/K+ ATPase) in the myocardium [5]. By inhibiting Na+/K+ ATPase, digoxin reverses the action of the sodium-calcium exchanger and causes an increase in the intracellular calcium concentration. Elevated intracellular calcium leads to increased myocardial contractility without increasing heart energy expenditure. Effective treatment for heart failure and atrial fibrillation can significantly reduce the risk of malignant arrhythmia. Despite the well-recognized efficacy, due to a relatively narrow therapeutic index of digoxin [6], adverse reactions are commonly observed. But there is still uncertainty about the risk of anemia associated with digoxin treatment.

In the present study, we retrieved clinical reports of atrial fibrillation and heart failure patients from the FDA Adverse Event Reporting System (FAERS), a pharmacovigilance system maintained by FDA to monitor the safety of marketed drug products. Adverse events were submitted to FAERS by healthcare professionals and patients on a voluntary basis. Over these years, the updated data of FAERS are released to the public periodically, which support drug safety research worldwide [7–9]. It used to take a great deal of effort to download and preprocess the raw data of FAERS. Fortunately, the openFDA project [10] was launched in June 2014 to provide convenient access to the FAERS reports in a structured and computer-readable format. This progress enabled us to efficiently retrieve adverse event reports during the period from 2004 to 2015, and examine the association between digoxin and anemia adverse reaction. In addition, we explored previously published evidences to extract a set of human genes interacting with digoxin. Functional enrichment analysis on these genes elucidated the potential toxicological mechanisms of digoxin-induced anemia.

## RESULTS

### Pooled analysis on reports of atrial fibrillation patients

**Figure 1:**
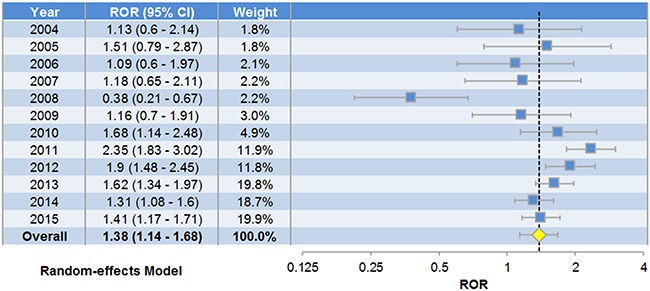
Forest plot of random-effects pooled analysis on anemia adverse events among atrial fibrillation patients The ROR of each year (the blue node) and the pooled ROR (the yellow node) along with 95% confidence intervals (the horizontal bars) are displayed.

**Figure 2:**
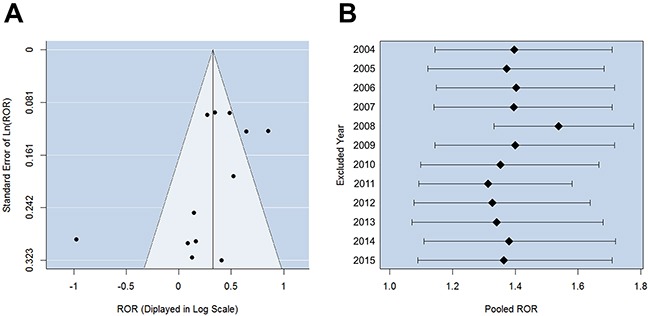
Examination of bias in the raw data of atrial fibrillation cases **(A)** Funnel plot for detecting reporting bias of anemia adverse events among atrial fibrillation patients. The result of Egger's linear regression test suggested the symmetry of funnel plot. **(B)** Sensitivity analysis for the association between digoxin treatment and anemia adverse reaction among atrial fibrillation patients. The RORs (the black nodes) and 95% confidence intervals (the horizontal bars) are displayed.

The analysis on atrial fibrillation patients involved 8142 cases exposed to digoxin and 67476 cases exposed to other drugs (see Materials and Methods, Supplementary Table 1). Case reports of different years were examined separately and combined into a pooled reporting odds ratio (ROR) [11]. The χ^2^-based Q test indicated significant heterogeneity among different years (P-value = 3.25×10^−6^, I^2^ = 76.08%), so a random-effects model was selected to pool the data (Appendix 1). The pooled ROR suggested that digoxin treatment was significantly concurrent with anemia adverse reaction (Figure 1, ROR = 1.38, P-value = 0.001). Pearson's Chi-square test (P-value = 4.06×10^−8^) and G-test (P-value = 7.90×10^−8^) also indicated significant association. To confirm whether the results were distorted by possible bias in the FAERS reports, we produced funnel plot and performed sensitivity analysis. Funnel plot is a scatterplot of treatment effect against a measure of study precision, whose asymmetric shape indicates systematic bias. Here we found no evidence of reporting bias in FAERS data, since the Egger's test (P-value > 0.05) validated the symmetry of funnel plot (Figure 2A). Sensitivity analysis was also performed to determine whether the data of a specific year would impact the overall results. Sequential omission of the data of individual years from 2004 to 2015 produced pooled RORs ranging from 1.31 to 1.54. The lower bounds of 95% CIs were constantly above 1.07, indicating that the pooled ROR was not unduly influenced by deleting the cases of any single year (Figure 2B).

### Pooled analysis on reports of heart failure patients

**Figure 3:**
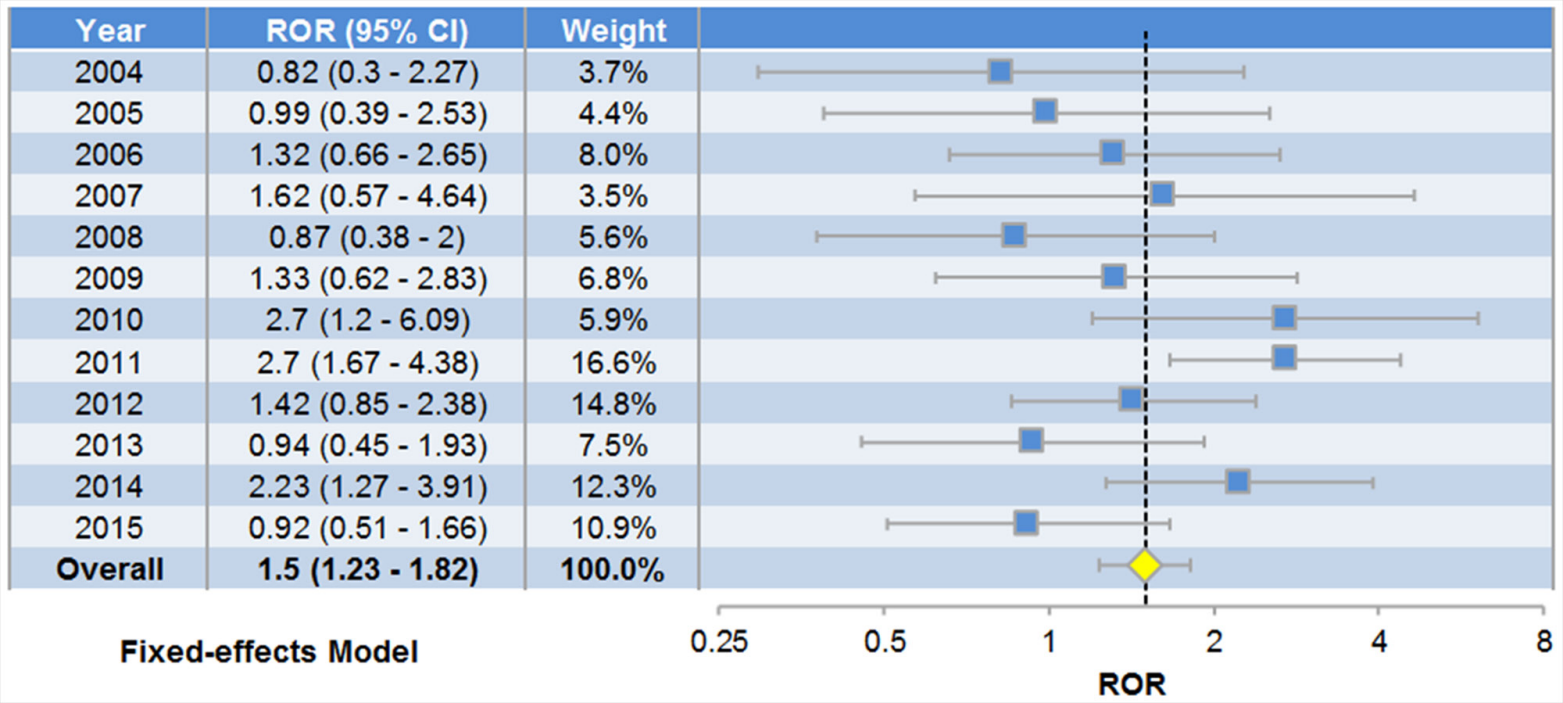
Forest plot of fixed-effects pooled analysis on anemia adverse events among heart failure patients The ROR of each year (the blue node) and the pooled ROR (the yellow node) along with 95% confidence intervals (the horizontal bars) are displayed.

**Figure 4:**
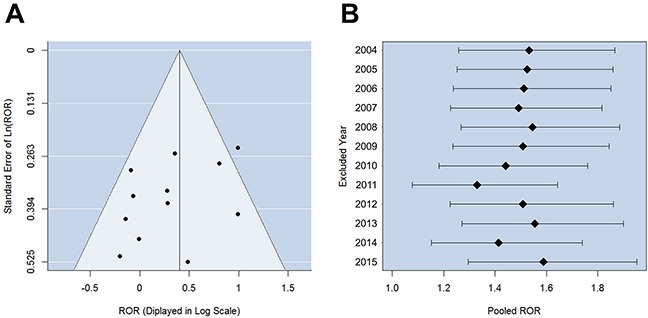
Examination of bias in the raw data of heart failure cases **(A)** Funnel plot for detecting reporting bias of anemia adverse events among heart failure patients. The result of Egger's linear regression test suggested the symmetry of funnel plot. **(B)** Sensitivity analysis for the association between digoxin treatment and anemia adverse reaction among heart failure patients. The RORs (the black nodes) and 95% confidence intervals (the horizontal bars) are displayed.

The reports of 2588 heart failure patients treated with digoxin and 13111 patients treated with other drugs were examined (Supplementary Table 2). No significant heterogeneity among different years was indicated by the χ^2^-based Q test (P-value = 0.067, I^2^ = 41.17%), so a fixed-effects model was selected. The combined effect size of different years brought in a pooled ROR = 1.50 (Figure 3), suggesting that digoxin treatment was significantly concurrent with anemia events among heart failure patients (P-value = 4.27×10^−5^). Pearson's Chi-square test (P-value = 0.003) and G-test (P-value = 0.003) also indicated significant association. No significant reporting bias was observed with P-value > 0.05 of Egger's test (Figure 4A). We also performed sensitivity analysis (Figure 4B) and found the omitted data of individual years caused no substantial change to the pooled ROR (ranging from 1.33 to 1.59). The lower confidence bounds of 95% CIs were always above 1.08.

### Analysis on genes interacting with digoxin

**Figure 5:**
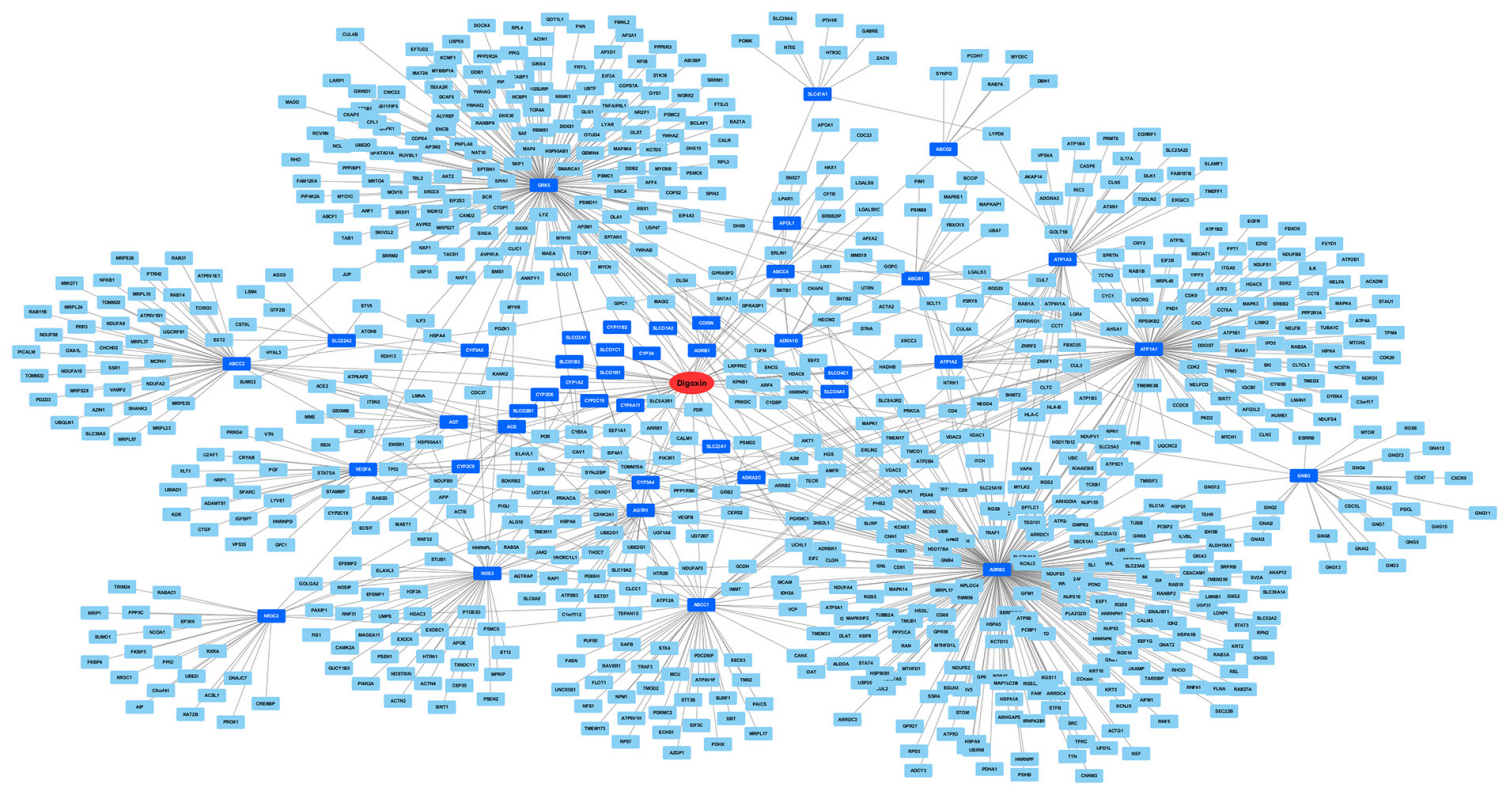
Visualization of drug-gene interaction network The red elliptic node represents the drug of interest (i.e., digoxin). The dark and light blue rectangular nodes represent genes directly and indirectly interacting with digoxin, respectively. The edges linking two nodes represent drug-gene or gene-gene interactions.

It is a well-known fact that drugs act on human body through the interaction with various proteins encoded by different genes [12]. In recent years, exploration of drug-gene interaction greatly improved our understanding to drug toxicity [13, 14]. To address this need, we primarily searched PharmGKB database and retrieved 42 genes directly interacting with digoxin based on curated literature review by PharmGKB experts. Next, we queried the BioGRID database and retrieved 779 genes interacting with the above 42 genes according to published experimental evidences. These 779 genes were regarded as indirectly associated with digoxin (See Materials and Methods). All thegenes and dogoxin were integrated into a network with 822 nodes (i.e., 821 genes and 1 drug) and 988 edges, which characterized the pharmacology of digoxin (Figure 5). To translate the network into biological insights, we further performed functional enrichment analysis for Gene Ontology (GO) biological processes [15]. First of all, compared with the background of human genome, the genes directly and indirectly interacting with digoxin were unsurprisingly enriched in a number of GO terms involved in cardiac function (Table 1). These GO terms well characterized the therapeutic effects of digoxin on heart disease. Beyond that, we also found significant enrichment in GO terms related to iron transport (Table 2), including ‘ferric iron transport’ (adjusted P-value = 1.63×10^−4^) and ‘iron ion transport’ (adjusted P-value = 2.29×10^−4^). This finding suggested that digoxin could profoundly interference with iron metabolism and increase the risk of anemia (see Discussion below).

**Table 1:** A portion of GO terms related to cardiac function are significantly enriched with digoxin-related genes

GO ID	GO term	Enrichment rate	Adjusted P-value	Overlap genes
GO:0008016	regulation of heart contraction	3.22	4.22E-08	CORIN, CTGF, ADRB1, DSG2, AGT, FLNA, SIRT1, JAK2, JUP, KCNE1, KCNJ3, KCNJ5, MDM2, ATP1A1, ATP1A2, ATP1A3, ATP1B1, ATP1B2, ATP1B3, NOS3, ATP2A2, ATP2B1, ATP2B3, ATP2B4, FXYD1, AVPR1A, PRKACA, ACE2, RGS2, SNTA1, SUMO1, CALM1, CAV1, CAV3, NUP155
GO:1903779	regulation of cardiac conduction	5.56	7.96E-08	CORIN, AGT, FLNA, ATP1A1, ATP1A2, ATP1A3, ATP1B1, ATP1B2, ATP1B3, ATP2A2, ATP2B1, ATP2B3, ATP2B4, FXYD1, PRKACA, ACE2, CALM1, CAV1
GO:0055117	regulation of cardiac muscle contraction	4.57	1.52E-05	CTGF, DSG2, FLNA, JUP, ATP1A1, ATP1A2, ATP1B1, ATP2A2, PRKACA, ACE2, RGS2, SUMO1, CALM1, CAV1, CAV3
GO:0086001	cardiac muscle cell action potential	4.52	3.52E-05	DSG2, FLNA, JUP, KCNE1, KCNJ3, KCNJ5, ATP1A1, ATP1A2, ATP1B1, ATP2A2, SNTA1, CAV1, CAV3, NUP155
GO:0002027	regulation of heart rate	3.78	7.21E-05	ADRB1, DSG2, AGT, SIRT1, JUP, KCNE1, KCNJ3, KCNJ5, MDM2, ATP2A2, AVPR1A, PRKACA, SNTA1, CALM1, CAV1, CAV3
GO:0003300	cardiac muscle hypertrophy	4.20	1.56E-04	CDK9, AGT, EZH2, MTOR, HTR2B, ATP2A2, ATP2B4, PPP3CA, PRKCA, RGS2, TTN, CAV3, CTDP1
GO:0035051	cardiocyte differentiation	3.03	5.80E-04	AGT, EGFR, MTOR, HNRNPU, LMNA, ARRB2, MYH10, MAPK1, MAPK3, PROX1, RXRA, TTN, VEGFA, CALR, CAV3, ACTN2, CTDP1
GO:0086005	ventricular cardiac muscle cell action potential	5.42	9.49E-04	DSG2, JUP, KCNE1, KCNJ3, KCNJ5, SNTA1, CAV1, CAV3

**Table 2:** A portion of GO terms related to iron transport are significantly enriched with digoxin-related genes

GO ID	GO term	Enrichment rate	Adjusted P-value	Overlap genes
GO:0015682	ferric iron transport	5.39	1.63E-04	TCIRG1, CLTC, ATP6V1H, ATP6V1A, ATP6V1B1, ATP6V1E1, TFRC, ATP6V0D1, RAB11B, ATP6V1F
GO:0006826	iron ion transport	4.35	2.29E-04	TCIRG1, CLTC, SLC39A14, ATP6V1H, ATP6V1A, ATP6V1B1, ATP6V1E1, TFRC, PICALM, ATP6V0D1, RAB11B, ATP6V1F

## DISCUSSION

Digoxin is widely used but has a relatively narrow therapeutic index. As a result, patients taking digoxin may be subjected to a number of adverse reactions. According to the drug label approved by FDA [16], digoxin is warned for anorexia, nausea, vomiting, visual changes, cardiac arrhythmias and some other safety risks. However, the potential of risk of digoxin-induced anemia has not been included in the official drug label. In this study, we retrieved a large number of reports from FAERS for data analysis. The results demonstrated that patients exposed to digoxin were significantly more likely to report anemia than non-digoxin controls. As far as we know, this is the first study that identifies the anemia risk of digoxin. Early awareness of the hematologic complication of digoxin will facilitate appropriate clinical management, such as supplementation of oral iron salts [17].

To better understand the underlying toxicological mechanisms of digoxin-induced anemia, we extracted data from public databases and constructed a drug-gene interaction network. A total of 821 human genes interacting with digoxin were investigated. Some of these genes were highly enriched in GO terms related to iron transport (as shown in Table 2). For instance, TFRC gene (i.e., transferrin receptor) encodes a cell surface receptor necessary for cellular iron uptake, which is essential for development of red blood cells [18]. And iron deficiency anemia is observed in a mouse model of missense mutation in TFRC [19]. As another example, PICALM gene (i.e., phosphatidylinositol binding clathrin assembly) interacts with TFRC as a critical regulator of transferrin receptor endocytosis [20]. Mutations in this gene are proved to be responsible for iron metabolism abnormalities in mouse model [21]. And PICALM knockout mice are found to develop iron deficiency anemia [22]. These evidences suggested that digoxin may disturb transferrin signaling and iron metabolism. A number of drugs, such as omeprazole [23] and carbimazole [24], have been reported to cause iron deficiency and anemia. Here we identified digoxin as another suspected drug. Experimental validation of digoxin-gene interactions will be required to elucid ate the underlying toxicological mechanisms of digoxin.

Despite of valuable findings in this study, some limitations of current results should still be kept in mind. First, all reports in FAERS were related to drug users affected by certain side effects, without healthy or unaffected control subjects. Referring to previous studies on FAERS [25, 26], the reports of digoxin were considered as cases, and the remaining reports of other drugs constituted potential controls. Since the controls were not exposed, it was reasonable to calculate the ROR by applying the principles of a case-control study. Second, since all reports were submitted to FAERS on a voluntary basis, some detailed information of patients, such as length of disease, drug dosage or drug combination, was missing in many reports [27]. Particularly, digoxin is widely used in combination with other drugs for heart conditions [28, 29]. Since FAERS data are based on spontaneous reports without rigorous examination, the results may be complicated by potential digoxin-drug interactions [30]. For that reason, large-sample and well-controlled trials will be required to investigate more factors involved in the safety risks of digoxin.

Taken together, anemia adverse events were significantly more reported by atrial fibrillation and heart failure patients taking digoxin than those taking other drugs. Therefore, great caution should be exercised in prescribing digoxin to patients with hematological conditions. Meanwhile, the genes interacting with digoxin were found to be significantly enriched in functional categories of iron transport, which pointed out the direction of future toxicological research.

## MATERIALS AND METHODS

### FAERS data extraction

The original reports of adverse events restored in the FDA Adverse Event Reporting System (FAERS) were queried through OpenFDA platform following the official instructions (https://open.fda.gov/api/reference/). The adverse events of each year between 2004 and 2015 were extracted separately. The adverse events concurrent with digoxin treatment were queried with the drug generic name “DIGOXIN”. The anemia adverse events were queried using the term “ANAEMIA”. According to the offical statement of FDA (https://www.fda.gov/Drugs/GuidanceComplianceRegulatoryInformation/Surveillance/AdverseDrugEffects/default.htm), the reports in FAERS are evaluated by clinical reviewers at FDA to monitor the safety of products after they are approved by FDA. If a potential safety concern is identified in FAERS, further evaluation is performed. Therefore, patients with pre-existed anemic conditions should have been excluded by FDA before data release. The reports of atrial fibrillation and heart failure patients were specified with drug indication index for “ATRIAL FIBRILLATION” AND “CARDIAC FAILURE”, respectively. The above terms were coded using MedDRA terminology (http://www.meddra.org/). Two investigators independently queried the data and the results were reviewed by a third investigator. Inconsistency was solved by discussion with the whole research team.

### Statistical analysis

A two-by-two contingency table was constructed with the FAERS data of each year. The subjects were classified by anemia adverse events (reported or not reported) and digoxin exposure (exposed or not exposed). The comparison between digoxin and other drugs in the two-by-two table was summarized by using the reporting odds ratio (ROR) [11]. An ROR greater than 1.0 indicated a higher risk of digoxin than other drugs. The RORs of different years were pooled together by using the ‘metafor’ package of R software (https://cran.r-project.org/web/packages/metafor/). The heterogeneity among different years was examined with the χ^2^-based Q test [31]. Q statistic is calculated as the weighted sum of squared differences between individual years. If there is great between-year heterogeneity, Q statistic is reported with a low P-value (i.e., P-value < 0.05). I^2^ statistic lies between 0% and 100% and represents the percentage of observed total variation across studies that is due to real heterogeneity rather than chance. It is calculated as I^2^ = 100% x (Q - df)/Q, where Q is the Q statistic and df the degrees of freedom. An I^2^ value over 50% indicates substantial heterogeneity. If there was no significant heterogeneity, a fixed-effects model (the Mantel-Haenszel method) was selected to pool the data. With fixed effects, data of individual years are considered to have been collected under similar conditions. Otherwise, a random-effects model (the DerSimonian and Laird method) was selected to pool the data, which admits data of individual years vary in a normal distribution. Pearson's Chi-square test and G-test were also performed with R software (version 3.2.5) to confirm the significance of association. The reporting bias was examined by performing Egger's linear regression test on the asymmetry of funnel plot [32]. P < 0.05 was considered to indicate statistically significant asymmetry. In addition, leave-one-out sensitivity analysis was performed to assess the robustness of the results.

### Drug-gene interaction network

The drug-gene network was constructed on the basis of drug-gene and gene-gene interactions. The digoxin-associated genes (i.e., genes directly interacting with digoxin) were retrieved from the PharmGKB (https://www.pharmgkb.org) database [33]. And the indirectly associated genes (i.e., genes indirectly linked with digoxin by interacting with digoxin-associated genes) were retrieved from the BioGRID database version 3.4.144 (https://thebiogrid.org) [34]. All the above interactions were integrated into a network structure by using the Cytoscape software, with was downloaded at the Cytoscape website (http://cytoscape.org/) and operated following the official manual. The biological function of the genes in this network was investigated by using the WebGestalt online tool (http://www.webgestalt.org/) [35]. This tool performs hypergeometric test and evaluates the enrichment for the GO terms in a specific gene list. As multiple terms/pathways were tested at the same time, the p-values of enrichment were adjusted using the Benjamini-Hochberg procedure. Adjusted p-value < 0.05 were selected as the threshold of significant enrichment.

## SUPPLEMENTARY MATERIALS TABLES


