# Supplementary material for: Digoxin-induced anemia among patients with atrial fibrillation and heart failure: clinical data analysis and drug-gene interaction network
Source: Oncotarget. 2017 Jun 16;8(34):57003–11. doi: 10.18632/oncotarget.18504 (PMC5593620; doi:10.18632/oncotarget.18504)
Supplement: Supplementary file 1 [file oncotarget-08-57003-s001.pdf]

Digoxin-induced anemia among patients with atrial fibrillation and heart failure: clinical data analysis and drug-gene interaction network

SUPPLEMENTARY MATERIALS

Supplementary Table 1: Summary of reports of atrial fibrillation patients

Year	Digoxin used anemia reported	Digoxin used anemia not reported	Digoxin not used anemia reported	Digoxin not used anemia not reported
2004	14	342	39	1080
2005	14	312	35	1175
2006	16	323	47	1033
2007	16	324	51	1214
2008	14	518	98	1366
2009	22	389	74	1514
2010	37	476	123	2666
2011	90	1153	286	8609
2012	82	818	450	8543
2013	141	806	1070	9936
2014	129	822	1194	10003
2015	132	1152	1265	15605

Supplementary Table 2: Summary of reports of heart failure patients

Year	Digoxin used anemia reported	Digoxin used anemia not reported	Digoxin not used anemia reported	Digoxin not used anemia not reported
2004	5	172	19	535
2005	6	180	22	655
2006	12	129	35	496
2007	5	134	15	652
2008	8	316	22	754
2009	10	198	27	710
2010	10	181	17	831
2011	31	287	46	1151
2012	21	247	75	1257
2013	9	185	76	1461
2014	17	174	74	1692
2015	13	238	140	2349
